# An Unusual Presentation of Barakat Syndrome: Gene Deletion at Chromosome 10p15

**DOI:** 10.1155/crin/8837745

**Published:** 2025-09-18

**Authors:** Matthew Satariano, Shaarav Ghose, Sergul Erzurum, Erdal Sarac

**Affiliations:** ^1^Department of Medicine, Northeast Ohio Medical University College of Medicine, Rootstown, Ohio, USA; ^2^Department of Internal Medicine, Mercy Health St Elizabeth Youngstown Hospital, Youngstown, Ohio, USA

**Keywords:** barakat, deafness, *GATA3*, genetic, HDRS, hypoparathyroidism, renal

## Abstract

Barakat syndrome, also known as HDR syndrome (HDRS), is an autosomal dominant genetic disease classically characterized by hypoparathyroidism (H), deafness (D), and renal disease (R). Less than 200 patients have been reported in the literature since it was first described in 1977 and has meanwhile been shown to have considerable genotypic variability. Barakat syndrome is usually caused by a mutation or knockout in *GATA3*, a zinc finger protein found on chromosome 10p14 which plays a role in embryologic formation of the central nervous system, thymus, auditory apparatus, kidney, and parathyroid glands. A spectrum of genetic variances in this gene has been related to HDRS, including both noncoding and coding regions with subsequent point mutations, wild-type protein disturbances, and haploinsufficiency. This case presents a 38-year-old female patient with recurrent urinary infections, hearing loss, and chronic kidney disease who underwent extensive laboratory, radiological, and genetic analysis which demonstrated a *GATA3* mutation in the 10p15 location. This specific genetic variability is currently absent on the gnomAD database, highlighting the rarity of the mutation. It is crucial to identify rare presentations of Barakat syndrome to allow for the best management, which often revolves around symptomatic management. HDRS prognosis is often determined by the progression of renal disease and thus should be the primary focus of the physician's care of the patient. This case contributes to the body of literature supporting the unique presentation and genetic variability of Barakat syndrome.

## 1. Introduction

Barakat syndrome, also known as HDR syndrome (HDRS), is a rare inherited disorder classically characterized by hypoparathyroidism (H), deafness (D), and renal disease (R). Since it was identified by Barakat et al. in 1977, fewer than 200 patients have been reported in the literature. Subsequent discoveries have unveiled additional cases outlining more associated clinical features including cognitive impairment, congenital cardiac defects, hypogonadotropic hypogonadism, polycystic ovaries, and retinitis pigmentosa [1, 2].

The underlying genetic cause of HDRS is associated with mutations in the *GATA3* gene, a zinc finger protein situated on chromosome 10p14. *GATA3* plays a role in embryologic formation of the central nervous system, thymus, auditory apparatus, kidney, and parathyroid glands [3, 4]. A spectrum of genetic variances in this gene have been related to HDRS, including both noncoding and coding regions with subsequent point mutations, wild-type protein disturbances, and haploinsufficiency [5]. Current guidelines propose confirming a diagnosis of HDRS in individuals exhibiting all three components (H, D, or R) or in those with two components along with a positive family history. Due to the high cost of genetic testing and genotypic variability, *GATA3* testing is optional to confirm a definitive diagnosis [5]. Given the rarity of Barakat syndrome, it is important to document the different genetic variances that cause the disorder as well as clinical presentation to best guide management and counseling. In this case report, we present a patient featuring a rare *GATA3* mutation situated at 10p15, exhibiting both recurrent urinary tract infections (UTIs) and hearing loss.

## 2. Case Report

A 38-year-old female with recurrent UTIs and episodes of acute kidney injury (AKI) triggered by dehydration consulted a nephrologist. Despite exhibiting a baseline serum creatinine level within normal limits at 1.1 mg/dL, her creatinine levels would rise to as high as 4 mg/dL during episodes of dehydration. The patient exhibited rapid recovery from these episodes of AKI following fluid administration. A voiding cystourethrogram (VCUG) was performed on the patient, yielding normal findings. Over the years, the patient was managed through regular scheduled check-ups and an emphasis on maintaining hydration through fluid intake to prevent episodes of AKI. The AKI episodes were diagnosed per Kidney Disease: Improving Global Outcomes (KDIGO) criteria.

**Figure 1:**
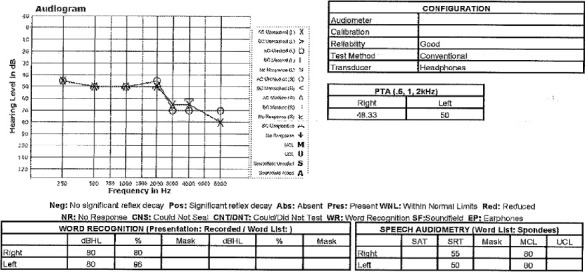
Audiology report.

Two decades later, the patient's chronic kidney disease (CKD) progressed to Stage 3b with proteinuria, believed to be associated with the recurrent episodes of AKI. Laboratory tests demonstrated an elevated creatinine of 1.52 mg/dL (which was previously 1.62 mg/dL and 1.60 mg/dL at 4 and 1 months prior, respectively) and normal parathyroid hormone levels (stable at 27 pg/mL from 4 to 1 month prior) (Table 1). A renal ultrasound was performed which displayed normal sized kidneys, preservation of the renal cortex, several 1–1.5 cm cysts, and renal calculi measuring up to 0.9 cm on the left kidney. Over the course of the patient's care, hearing impairment was noted with the onset at 59 years of age. The presence of a familial history of hearing loss in both the patient's father and biological daughter as well as chronic renal dysfunction led to an increased suspicion of Alport syndrome. The nephrologist recommended a hearing evaluation resulting in a diagnosis of sensorineural hearing loss. More specifically, the pure tone testing showed symmetric, moderate-to-severe sensorineural sloping hearing loss in both the right and left ears, and the word recognition was rated as good in the right ear and excellent in the left. An image of this report is included (Figure 1).

**Figure 2:**
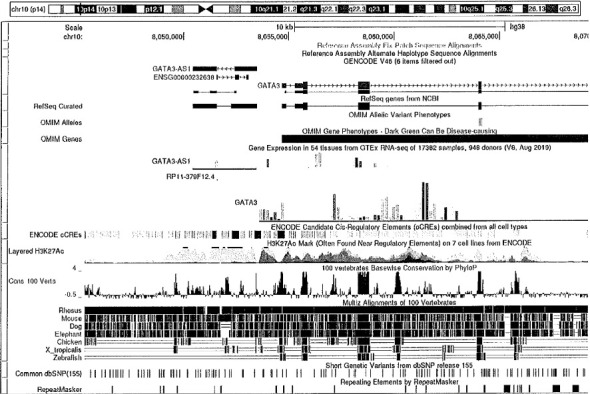
*GATA3* genetic test.

Given the patient's clinical manifestations, the nephrologist recommended genetic testing revealing a heterozygous *GATA3* whole gene deletion of all coding regions which encompassed the genetic region exons 2–6 (exon 1 is noncoding) on 10p15 (coordinates include a start position of 8,045,378 and end position of 8,075,198) [6]. Based on the guidelines provided by the American College of Medical Genetics and Genomics, this would be classified as pathogenic [7]. A specific image of this genetic test is included in Figure 2. This genetic anomaly is linked with Barakat syndrome. The specific genetic test utilized was Renasight which is a next-generation sequencing test [8]. Although the initial suspected diagnosis was Alport syndrome, the presence of the heterozygous *GATA3* whole gene deletion increased the possibility of Barakat syndrome, and the lack of ocular abnormalities and hematuria made Alport syndrome less likely. Moreover, genetic testing did not reveal any pathogenic variants in the COL4A3, COL4A4, or COL4A5 genes which are typically associated with Alport syndrome. DiGeorge syndrome 2 (DGS2) was also included in the differential diagnosis due to its variable presentation, which can involve sensorineural hearing loss and renal anomalies. Considering the patient's genetic analysis, a positive family history, and the presence of two phenotypical diagnostic criteria (deafness and renal disease), a diagnosis of Barakat syndrome was established. While next-generation sequencing confirmed the deletion in our case, chromosomal microarray analysis (CMA) is also a valuable tool for detecting *GATA3* deletions involving chromosome 10p15. The patient received comprehensive education regarding the diagnosis and was strongly advised for genetic testing for the patient's family members, although they declined. A DEXA scan obtained in 2024 displayed mild osteopenia with a lowest T score of −1.7, ruling out osteoporosis. Additionally, specific measures including continued nephrology follow-up, hydration counseling, annual audiologic assessments, conservative management of nephrolithiasis, and osteoporosis prevention strategies were undertaken for the patient.

## 3. Discussion

This case report is a unique presentation of a whole *GATA3* gene deletion located at chromosome 10p15 associated with recurrent early onset UTI, hearing loss, and normal parathyroid hormone levels. This autosomal dominant genetic disease is extremely rare, with less than 200 identified patients in the literature [1]. Its phenotypic and genotypic variability make it rather challenging to identify. Late-onset presentation in this patient with sensorineural hearing loss first recognized at an age of 59 years is unusual for HDRS and may be explained by variable expressivity, incomplete penetrance, and compensatory physiological mechanisms that delay the onset of symptoms. Additionally, gradual decline in renal function over decades may mask the underlying syndrome until other features appear. The normal parathyroid hormone levels in this patient could reflect either incomplete penetrance of the hypoparathyroidism phenotype or secondary regulatory effects from CKD. Notably, this variant is currently absent on the gnomAD database, which shows rarity of the mutation. The patient's genetic testing results for the *GATA3* gene are demonstrated by Table 2. It is important to recognize various presentations to allow for the best detection and treatment of Barakat syndrome. One recent study identified a novel frameshift mutation, P227Afs, in *GATA3* which led to the loss of zinc finger structures and subsequent reduced transcriptional activity of the gene [9]. An additional study discovered a haploinsufficiency on 10p13-10p14, a more proximal region on *GATA3*, that presented with DiGeorge-like features and bilateral deafness, but without other HDRS characteristics [10]. Another study discovered deletions on both 10p14 and 10p13-14 in a patient who presented with both features of HDRS and DGS [11]. One recent case report from 2023 identified Barakat syndrome in a patient with a *GATA3*:c.916C > T nonsense mutation on chromosome 10p14 [2]. Interestingly, one review of HDRS did not identify a *GATA3* mutation in 9.4% of patients [5].

Patients with HDRS typically present with hypocalcemia secondary to hypoparathyroidism. In fact, a review of 180 patients with HDRS found 93.3% of individuals had hypoparathyroidism [5], 96.7% of the patients had hearing loss, and 72.2% had renal defects [5]. The renal defects and hearing abnormalities are often identified in the neonatal period by routine ultrasonography and hearing screenings, respectively. For example, one case report presented a 24-day-old girl who had an abnormal newborn hearing screening examination, hypoparathyroidism (parathyroid levels as low as 7 pg/mL), hypocalcemia (calcium levels of 6.1 mg/dL), and a family history of bilateral sensorineural hearing loss and hypoparathyroidism who was subsequently diagnosed with HDRS [12]. These phenotypic and genetic variations raise the diagnostic complexity of HDRS in establishing a definitive diagnosis.

Management typically revolves around symptomatic treatment. Hypocalcemia associated with HDRS is often managed in the outpatient setting. However, individuals with severe calcium imbalances are at risk for cardiomyopathy, laryngospasm, tetany, and seizures [5]. Therefore, it is important to monitor electrolyte levels as well as electrocardiogram results for those at risk. Hypercalciuria should be monitored by collecting serial urinary calcium levels. Hypoparathyroidism can be managed with intravenous calcium gluconate or oral calcium and calcitriol depending on the severity of the findings. Sensorineural deafness is typically detected on neonatal screening exams and should be addressed with early intervention such as cochlear implantation [5]. HDRS prognosis is often determined by the progression of renal disease and thus should be a primary focus for the physician's care for the patient. In some cases, renal transplantation and dialysis are the only treatment options available [5]. In clinical practice, it is essential to remain alert for recurrent episodes of AKI with rapid recoveries coupled with hearing loss. This proactive approach can aid in the early diagnosis of Barakat syndrome and mitigate potential complications. Additionally, some literature suggests Barakat syndrome workup in patients who present with apparent idiopathic hypoparathyroidism [13]. In summary, we detail a case of HDRS featuring a rare *GATA3* mutation at 10p15, aiming to enrich the sparse literature and enhance guidance in managing and counseling the diverse variations of this uncommon genetic disorder.

## 4. Conclusion

The literature on HDRS remains sparse, with fewer than 200 cases reported. This case adds to the growing body of knowledge by highlighting a unique clinical presentation and a distinct genetic mutation, underscoring the genotypic and phenotypic variability of the disease. Key takeaways from this case include the importance of considering HDRS in patients with concurrent renal disease and sensorineural hearing loss, particularly when there is a familial history of similar findings. Additionally, the identification of a rare *GATA3* mutation emphasizes the need for continued genetic exploration in rare syndromes with overlapping features. Future research should focus on further elucidating the full spectrum of HDRS's clinical manifestations, the role of *GATA3* mutations in disease pathogenesis, and the development of standardized diagnostic and management protocols for affected individuals and their families.

## Figures and Tables

**Table 1:** Renal function and metabolic panel results.

Creatinine	1.52 mg/dL
Glomerular filtration rate	38 mL/min
Uric acid	5.6 mg/dL on allopurinol 200 mg daily
Calcium	6.1–9.2 mg/dL
Phosphorus	3.2–4.7 md/dL
Parathyroid hormone	27 pg/mL
Protein/creatinine ratio	0.44 g/day
Albumin/creatinine ratio	168 mg/L
25-Hydroxy vitamin D level	56 ng/mL
BUN	25 mg/mL
Sodium	140 mmol/L
Alkaline phosphatase (ALP)	93 U/L
Magnesium	1.7 mg/dL
Renal sonography impression	Suspect multiple bilateral small nonobstructing renal calculi and bilateral simple renal cysts

**Table 2:** Genetic testing results for *GATA3* gene.

Gene	Coordinates	Condition(s)	Inheritance	Variant(s)	Zygosity	Classification
GATA3	Start position: 8,045,378 End position: 8,075,198	Hypoparathyroidism, sensorineural deafness, and renal dysplasia	Autosomal dominant	Apparent whole gene deletion	Heterozygous	Pathogenic
